# Patient-specific primary and pluripotent stem cell-derived stromal cells recapitulate key aspects of arrhythmogenic cardiomyopathy

**DOI:** 10.1038/s41598-023-43308-2

**Published:** 2023-09-27

**Authors:** Angela Serena Maione, Viviana Meraviglia, Lara Iengo, Martina Rabino, Mattia Chiesa, Valentina Catto, Claudio Tondo, Giulio Pompilio, Milena Bellin, Elena Sommariva

**Affiliations:** 1 Unit of Vascular Biology and Regenerative Medicine, Centro Cardiologico Monzino IRCCS, 20138 Milan, Italy; 2 Department of Anatomy and Embryology, Leiden University Medical Center, 2333 ZC Leiden, The Netherlands; 3 Bioinformatics and Artificial Intelligence Facility, Centro Cardiologico Monzino IRCCS, 20138 Milan, Italy; 4 Department of Electronics, Information and Biomedical Engineering, Politecnico di Milano, 20133 Milan, Italy; 5 Department of Clinical Electrophysiology and Cardiac Pacing, Centro Cardiologico Monzino IRCCS, 20138 Milan, Italy; 6 Department of Biomedical, Surgical and Dental Sciences, Università Degli Studi Di Milano, 20122 Milan, Italy; 7 Department of Biology, University of Padua, 35121 Padua, Italy; 8 Veneto Institute of Molecular Medicine, 35129 Padua, Italy

**Keywords:** Mechanisms of disease, Heart stem cells, Pluripotent stem cells, Cardiomyopathies

## Abstract

Primary cardiac mesenchymal stromal cells (C-MSCs) can promote the aberrant remodeling of cardiac tissue that characterizes arrhythmogenic cardiomyopathy (ACM) by differentiating into adipocytes and myofibroblasts. These cells’ limitations, including restricted access to primary material and its manipulation have been overcome by the advancement of human induced pluripotent stem cells (hiPSCs), and their ability to differentiate towards the cardiac stromal population. C-MSCs derived from hiPSCs make it possible to work with virtually unlimited numbers of cells that are genetically identical to the cells of origin. We performed in vitro experiments on primary stromal cells (Primary) and hiPSC-derived stromal cells (hiPSC-D) to compare them as tools to model ACM. Both Primary and hiPSC-D cells expressed mesenchymal surface markers and possessed typical MSC differentiation potentials. hiPSC-D expressed desmosomal genes and proteins and shared a similar transcriptomic profile with Primary cells. Furthermore, ACM hiPSC-D exhibited higher propensity to accumulate lipid droplets and collagen compared to healthy control cells, similar to their primary counterparts. Therefore, both Primary and hiPSC-D cardiac stromal cells obtained from ACM patients can be used to model aspects of the disease. The choice of the most suitable model will depend on experimental needs and on the availability of human source samples.

Arrhythmogenic cardiomyopathy (ACM) is a rare heart disease, mostly inherited as an autosomal dominant trait and is largely attributable to mutations in genes encoding cardiac desmosomes. It is characterized by the replacement of the myocardium with fibro-fatty tissue, leading to malignant ventricular arrhythmias, heart failure and high risk of sudden death. The disease predominantly involves the right ventricle; however, left and biventricular involvement have also been reported^1^.

The human heart is composed of several cell types where 30% are cardiomyocytes and 70% are non-myocytes^2–4^. Among the latter, cardiac mesenchymal stromal cells (C-MSCs) are predominant and play critical roles in maintaining a physiologic cardiac homeostasis and contributing to cardiac repair in pathological conditions^5^. C-MSCs isolated from individuals diagnosed with ACM are known to contribute to the fibro-adipose remodeling of the patients’ hearts^6^. In addition, primary ACM C-MSCs, directly obtained from biopsy specimens of ACM patients, represent a reliable in vitro tool for ACM mechanistic studies due to their ability to differentiate into adipocytes and myofibroblasts^6–10^.

These cells can be easily amplified and maintained in vitro, carry the genetic background of the patient and are influenced by the in vivo cardiac pathogenic environment of origin. However, the use of primary C-MSCs is accompanied by some limitations such as: (1) the availability of fresh cardiac biopsy specimens; (2) the limited number of cells isolated from cardiac biopsies; (3) the limited number of in vitro passages that can be carried out, as occurs for all primary cells.

Thanks to the advances in inducted pluripotent stem cell (hiPSC) technology, limitations associated with primary cell manipulation have been overcome. HiPSCs represent a useful and versatile tool to study cardiomyopathies such as ACM, since they can replicate almost indefinitely, are genetically identical to the cells of origin, and can differentiate into different cell types, including cardiac stromal cells. Moreover, CRISPR/Cas9 technology is widely applied to correct the pathogenic mutation in patient-derived hiPSCs and generate isogenic corrected lines with the same genetic background, thus reducing biological variability, and allowing researchers to study variant-specific effects^11^.

The present study compares for the first time hiPSC-derived stromal cells (hiPSC-D) and primary stromal cells (Primary), highlighting the feasibility of using hiPSC-D as an in vitro model to study ACM, based on their homology with Primary cells. Overall, we (1) provided evidence that cardiac hiPSC-D express mesenchymal surface markers and are able to differentiate into osteoblasts and chondrocytes (2) demonstrated that cardiac hiPSC-D express desmosomal genes and proteins (3) validated the propensity of ACM hiPSC-D to accumulate more lipid and collagen compared to healthy control cells (4) confirmed, through a transcriptomic profiling, the high homology of hiPSC-D and Primary cells.

## Results

### HiPSC-D and Primary cells express similar mesenchymal markers and ability to differentiate into chondrocytes and osteocytes

In order to understand the similarities and differences between Primary and hiPSC-D stromal cells, we obtained the two cell types as follows. Primary cells were derived from right ventricle biopsies of ACM patients and healthy controls (HC; detailed in Table S1). Cardiac stromal cells from hiPSCs, reprogrammed from skin fibroblasts of the same patient (here called ACM patient 1) and healthy control subjects, were differentiated as previously described^12,13^ (as detailed in Table S2).

**Figure 1:**
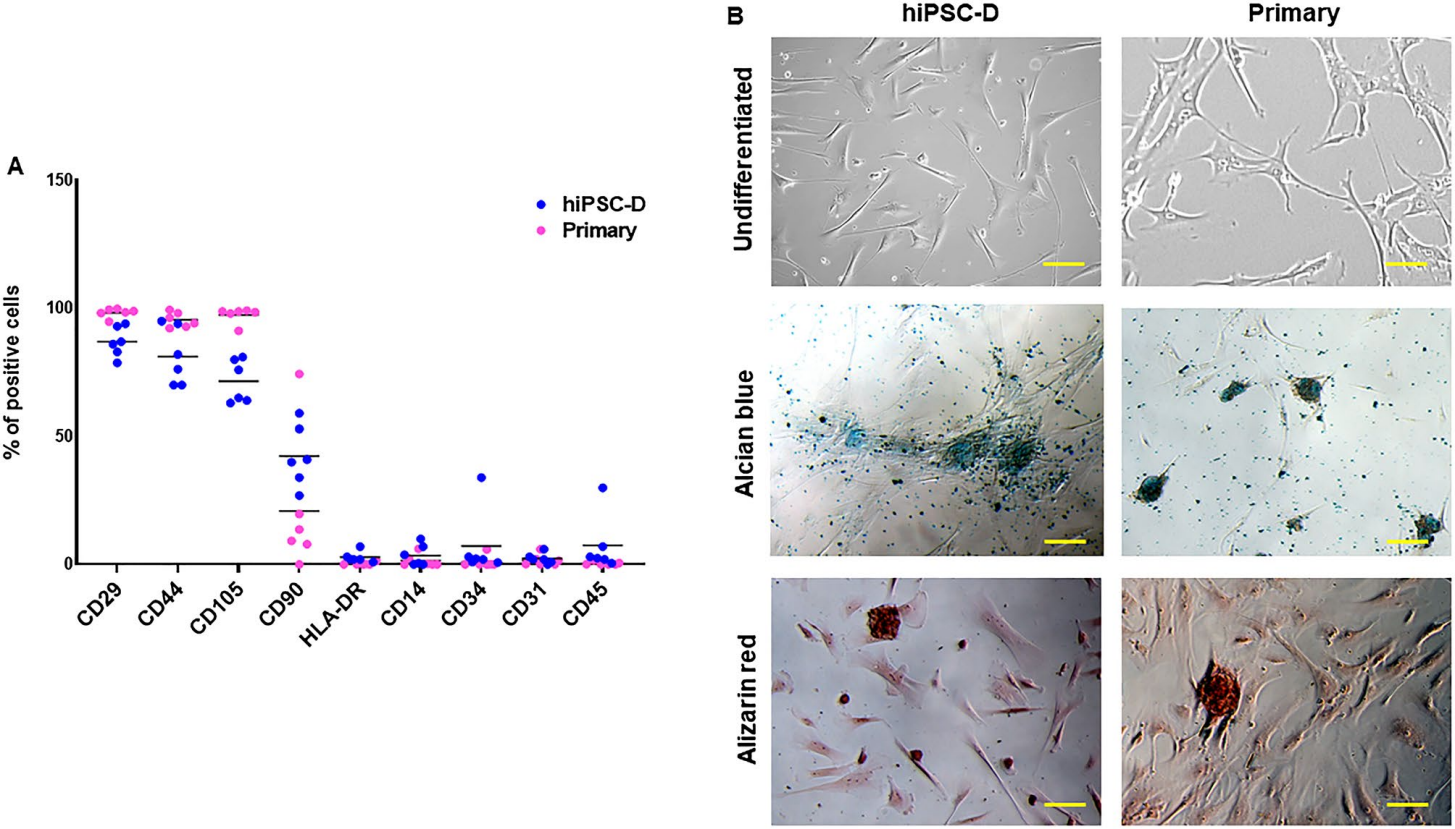
Expression of mesenchymal markers and chondrogenic-osteogenic commitment. (**A**) FACS analysis of hiPSC-D and Primary cells cultured in growth medium and stained with FITC/PE/APC-conjugated antibodies (CD29, CD44, CD105, CD90, HLA-DR, CD14, CD34, CD31 and CD45). The means FITC/PE/APC positive cells are shown as a comparison of hiPSC-D and Primary cells (including both HC and ACM patients) (n = 6 hiPSC-D vs. Primary cells). (**B**) Representative images of hiPSC-D and Primary cells: undifferentiated/unstained cells (top panels); Alcian blue (middle panels) and Alizarin red (bottom panels) stainings of cells cultured for 3 days in chondrogenic and osteogenic media, respectively. Magnification is 10X and the scale bar indicates 100 μm.Data information: mean.

To characterize hiPSC-D, we firstly investigated the positivity for specific mesenchymal surface markers as CD29, CD44 and CD105. We confirmed that hiPSC-D highly expressed CD29, CD44 and CD105 (Fig. 1A; S1) as previously shown for cardiac mesenchymal stromal cells (Primary)^6^. Variable levels of CD90 positive cells were observed for hiPSC-D stromal cells population (Fig. 1A; S1) as occurred for the heterogeneous population of Primary stromal cells^14^.

We validated the low expression of CD14, CD45, CD34, CD31 as hematopoietic and endothelial markers (Fig. 1A; S1). Moreover, HLA-DR, a marker of alloreactivity, was not detected in hiPSC-D, which was similarly reported in primary cells.^6,9^ (Fig. 1A; S1).

Primary stromal cells, isolated from different tissues, retain a residual plasticity and are able to differentiate towards mesenchymal lineages, under proper in vitro stimulation^6,7,15,16^. Therefore, we tested the differentiation potential of Primary and hiPSC-D cells by culturing them in specific culture conditions. As shown in Fig. 1B, both Primary and hiPSC-D cells were responsive to the differentiation induction when cultured on plastic and treated with chondrogenic or osteogenic medium, showing a positive reaction to Alcian blue and Alizarin red staining, respectively.

Overall, we observed a similar pattern for all the surface markers screened in hiPSC-D and Primary^6^ stromal cells and we also validated hiPSC-D’s ability to differentiate into chondrocytes and osteocytes, the results of which were similar to Primary cells.

### HiPSC-D cardiac stromal cells express proteins of the desmosome as Primary stromal cells

**Figure 2:**
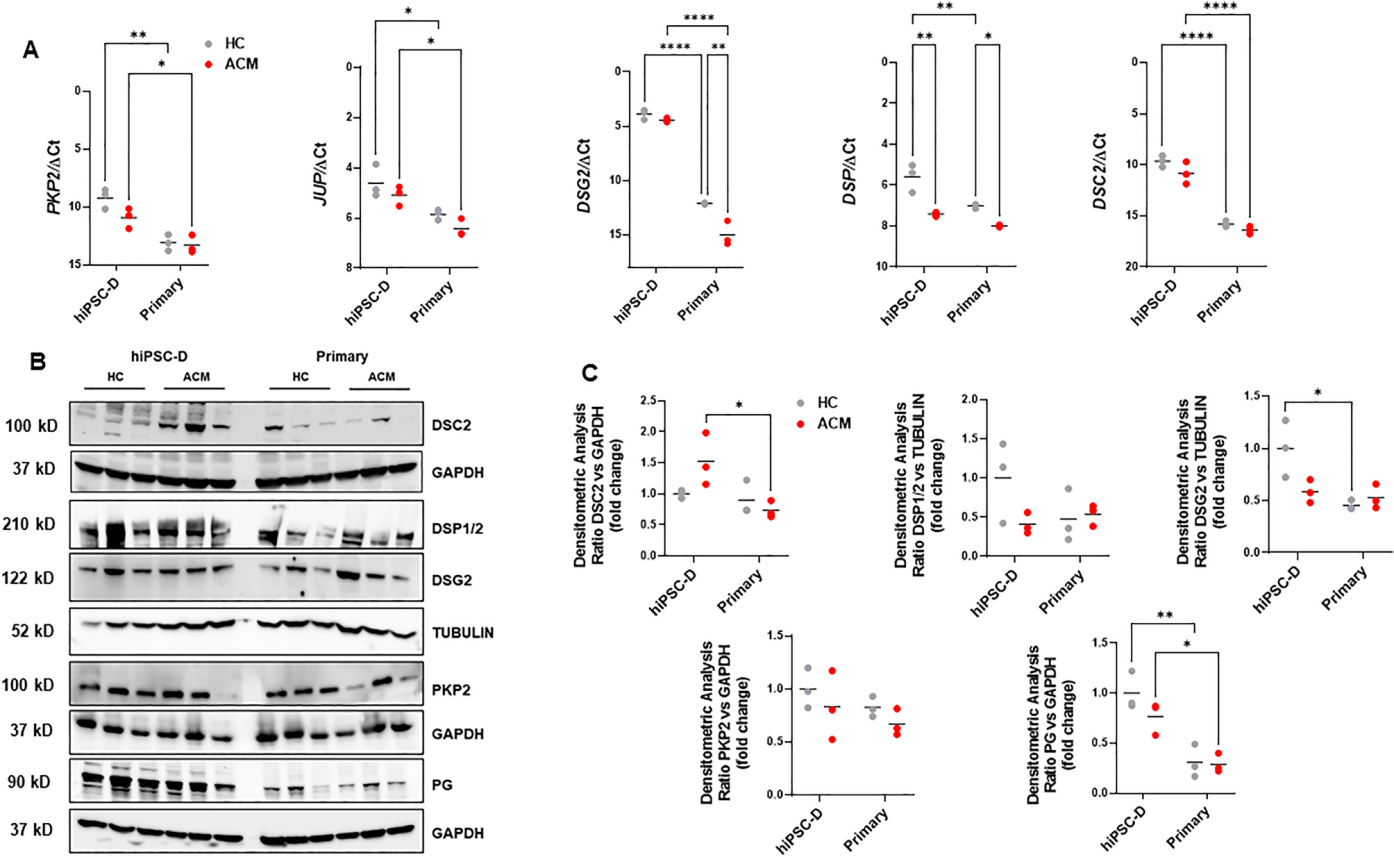
Expression of desmosomal gene and proteins. (**A**) Expression of *PKP2 JUP, DSG2, DSP* and *DSC2* in total RNA extracts of hiPSC-D and Primary cells from HC subjects and ACM patients cultured in growth medium. *GAPDH* was used as house-keeping gene and qRT-PCR data are presented as the genes threshold cycles (Ct) with respect to the housekeeping gene *GAPDH* (ΔCt) (n = 3 biological replicates; Two-way ANOVA and Tukey’s post-test). (**B**) Representative images of WB analysis of proteins extracted from hiPSC-D and Primary cells from HC and ACM individuals cultured in growth medium, hybridized with specific antibodies for desmosomal proteins (anti-DSC2, anti-DSP, anti-DSG2, anti-PKP2 and anti-PG). Immunostaining of the housekeeping GAPDH or TUBULIN is shown for normalization. (**C**) Densitometric analysis of desmosomal protein levels, normalized on GAPDH, has been shown as fold change respect to HC hiPSC-D in the graphs (*n* = 3 biological replicates; Two-way ANOVA and Tukey’s post-test). Data information: mean. *P < 0.05, **P < 0.01 and **** P < 0.0001.

We have previously described that the expression of desmosomal genes is not limited to cardiomyocytes^17,18^. They are expressed in other cardiac cell types such as Primary stromal cells^6^. qRT-PCR analysis showed that the desmosomal genes *PKP2*, *DSG2*, *DSP* and *DSC2* (Fig. 2A) were detectable in hiPSC-D cells. Western blot analysis confirmed qRT-PCR results, showing that PKP2, PG, DSG2, DSP and DSC2 (Fig. 2B,C; S2–S5) proteins were expressed in hiPSC-D stromal cells.

As for the comparison is between hiPSC-D and Primary, desmosomal genes and proteins were expressed at higher levels in hiPSC-D compared to Primary cells (Fig. 2).

In the case of cells obtained from patients with ACM and HC, DSP gene expression was significantly reduced in ACM as compared to that in HC, both in Primary and hiPSC-D cell populations. *DSG2* gene expression was significant reduced only in Primary cells. A general trend is evident of lower expression of desmosomal proteins in ACM cells, respect to HC, in hiPSC-D as is Primary cells, as previously reported^6^.

### HiPSC-D cardiac stromal cells from patient with arrhythmogenic cardiomyopathy show increased propensity to accumulate lipid droplets and collagen than control cells

**Figure 3:**
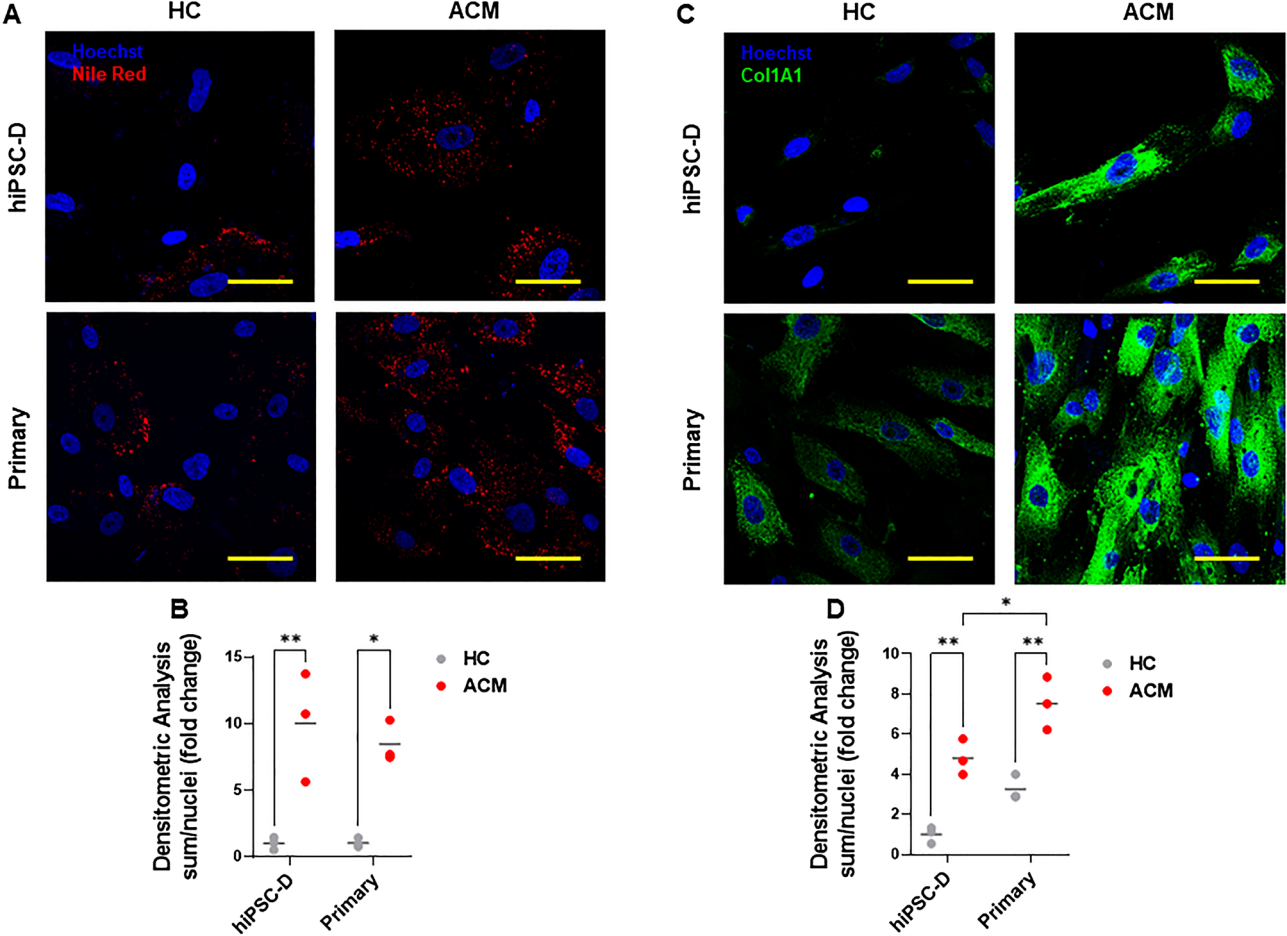
ACM cardiac stromal cells show fibro-adipogenic differentiation. (**A**) Representative images of Nile Red staining of hiPSC-D and Primary cells from HC subjects and ACM patients cultured in adipogenic medium for 3 days. Nuclei are stained with Hoechst 33,342. Magnification is 40X and the scale bar indicates 50 μm. (**B**) Quantification of Nile Red has been shown as fold change compared to HC hiPSC-D in the graphs (n = 3 HC hiPSC-D vs. ACM hiPSC-D vs. HC Primary vs. ACM Primary; Two-way ANOVA and Tukey’s post-test). (**C**) Representative images of immunostaining for COL1A1 of hiPSC-D and Primary cells (HC and ACM) cultured in low serum (2%) growth medium and stimulated with TGF-β1 for 3 days. Nuclei are stained with Hoechst 33,342. Magnification is 40X and the scale bar indicates 50 μm. (**D**) Quantification of COL1A1 has been shown as fold change compared to HC hiPSC-D in the graphs (n = 3 HC hiPSC-D vs. ACM hiPSC-D vs. HC Primary vs. ACM Primary; Two-way ANOVA and Tukey’s post-test). Data information: mean. *P < 0.05 and **P < 0.01.

Primary stromal cells are a source of adipocytes in ACM hearts and ACM-derived Primary stromal cells are able to differentiate in vitro into adipocytes^6^. Therefore, we tested the capability of hiPSC-D to differentiate into adipocytes following induction of adipogenic differentiation. HiPSC-D from HC and ACM were cultured in adipogenic conditions for three days, after which immunofluorescence analysis was performed to evaluate the lipid accumulation. Nile Red staining revealed that ACM hiPSC-D accumulated lipid droplets at a significantly higher amount than HC hiPSC-D cells; the levels were comparable to Primary cell ones (Fig. 3A,B).

Based on our recent findings demonstrating that Primary stromal cells contribute to fibrotic accumulation in ACM heart^7^, we analysed collagen deposition in hiPSC-D cells cultured in pro-fibrotic medium for three days. Immunofluorescence analysis revealed that the production of collagen I was higher in ACM hiPSC-D cells than in HC hiPSC-D cells, which was similarly reported for ACM Primary cells (Fig. 3C,D).

The expression of fibro-adipose genes after pro-adipose and pro-fibrotic differentiations was in line with the phenotypic results (Fig. S6).

### Transcriptomic analysis reveals a high degree of similarity between hiPSC-D and Primary cardiac stromal cells

**Figure 4:**
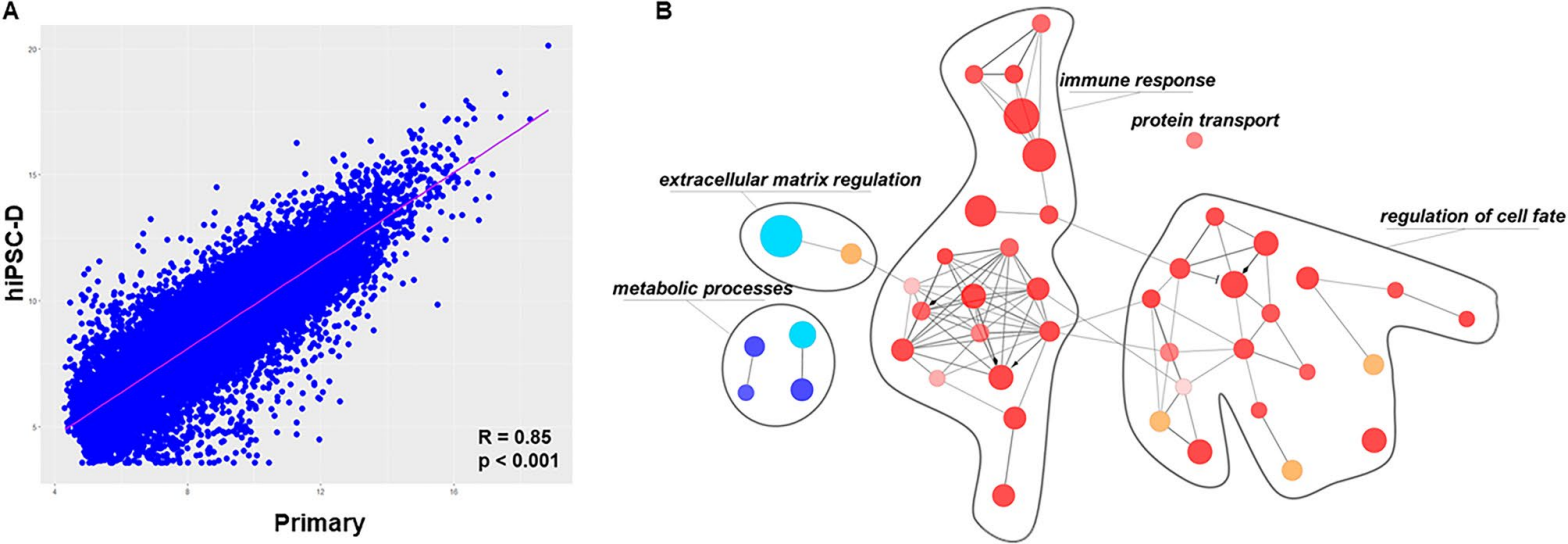
Transcriptomic profiling of Primary and hiPSC-D cells. (**A**) Scatter plot showing the relationship between Primary (x-axis) and hiPSC-D (y-axis) gene expression. Blue dots represent the gene, while purple line denotes the correlation trendline. (**B**) Pathways analysis highlighting the most significant processes associated to up-regulated genes in Primary cells compared to hiPSC-D (orange and red nodes; logFC > 2) and down-regulated genes (light blue and blue nodes; logFC < 2). The node size reflects the proportion of genes associated to the process while the node colour denotes the direction of the association: a node is blue when more than 60% of associated genes are down-regulated; a node is light blue when the proportion of down-regulated genes is between 50 and 60%; a node is red when more than 60% of associated genes are up-regulated; and, a node is orange when the proportion of down-regulated genes is between 50 and 60%.

To extend the characterization of differences and similarities between hiPSC-D and Primary cells, we obtained transcriptomic profiling of both cell types in basal culture conditions (Primary: GSE233780^19^; iPSC-D: GSE234504) and performed a standard correlation analysis, comparing the average gene expression levels in both conditions. As expected, we proved a strong association (*R* = 0.85, *p* < 0.001; Fig. 4A) between transcriptional expressions. This correlation was even more evident when comparing Primary cells from ACM *PKP2* mutated patient and hiPSC-D stromal cells obtained from the same patient (*R* = 0.98, *p* < 0.001; Fig. S7). Furthermore, selecting only those genes with a |logFC|> 2 between the two cell types, we identified a number of pathways enriched in hiPSC-D (red nodes, Fig. 4B) or in Primary stromal cells by GSEA (blue nodes, Fig. 4B). The pathways related to immune response and regulation of cell fate (fully listed in Table S3) were mostly associated with Primary stromal cells (Fig. 4B). Instead, the most representative pathways enriched in hiPSC-D stromal cells are related to metabolic processes and extracellular matrix regulation (fully listed in Table S3; Fig. 4B).

## Discussion

Cardiac stromal cells are useful tools for cardiac disease modelling. There are advantages and disadvantages associated with primary and hiPSC-D cells. Primary stromal cells better represent the patient’s cardiac microenvironment at the specific stage of the disease when the biopsy is taken. However, Primary cells are limited in amplification potentials, while hiPSC-D cells can be differentiated from hiPSCs virtually unlimitedly. HiPSCs can be genetically modified, for instance to correct the pathogenic mutation; thus hiPSC-D can be derived from the isogenic control, without the main cause of the disease. This is not virtually impossible in primary cells due to its limited life-frame. The material source of the cells is very different, being much more accessible those for hiPSC-D cells (nearly any somatic cell type). However, they suffer the possibility of introducing genetic mistakes in the long frame of their reprogramming, prolonged culture, differentiating processes, and the inevitability of modifying their epigenetic memory^20^. In addition, the cost of production, maintenance in culture and differentiation are still elevated and these processes are time-consuming.

Our work demonstrates that the two cell types are eventually very similar with comparable morphology. We notice a smaller volume of hiPSC-D stromal cells, compared to the Primary cells. This may be due to a certain degree of immaturity of hiPSC-D compared to their primary counterpart, which has a wider heterogeneity of maturity towards different fates. The two populations of cells display typical characteristics of MSCs^21^, which include being able to differentiate towards osteogenic, chondrogenic, adipogenic and myofibroblast lineages. Moreover, they are characterized by similar surface markers (variability within 10%), including variable expression of CD90, as described for the cardiac population of MSCs^22^, with the exception of the percentage of cells expressing CD105, which is higher in the Primary cells. Literature reports describe a variability in the CD105 expression in different MSC populations. In particular, CD105 negative cells are described as being more immunosuppressive, and with altered differentiation capability, including lower TGFβ signalling^23,24^.

We noticed a general trend of higher expression of desmosomal genes and proteins in hiPSC-D stromal cells compared to their primary counterpart, independent of the genotype (ACM or HC). We speculate that higher expression of desmosomal genes in hiPSC-D cells might be related to their tissue of origin, as hiPSCs were generated from skin fibroblast biopsy. Since the skin is one of the organs with the most abundant desmosomes, we hypothesize that hiPSC-D cells might retain a certain epigenetic memory of the material source^25^. In addition, a lower expression, even if not statistically significant, was confirmed for PKP2 in the ACM lines of both cell types, thus corroborating one of the characteristics of ACM stromal cells^6,12^.

Independently from their origin, ACM cells were shown to recapitulate the high propensity to adipogenesis and collagen synthesis, when properly stimulated, in comparison with their HC counterpart. However, ACM Primary cells treated with TGFβ showed the ability to produce higher collagen than the hiPSC-D ones. This could be due to the effect of the memory of their previous in vivo microenvironment stimulation, possibly causing a step forward in their myofibroblast fate^7^.

Transcriptomic analysis has also shown comparable results between the cells form different sources, with few pathways enriched in the Primary stromal cells, mostly linked to the immune response and regulation of cell fate. This finding would corroborate the idea that an inflammatory stimulus received in vivo in the cardiac tissue might activate an innate immunity in the stromal cells, thus potentially driving cell differentiation fates^26–30^. The fact that the metabolic processes are different in the two cell types also hints to slightly diverse stage of maturation^31,32^.

In conclusion, we demonstrated that both Primary and hiPSC-D stromal cells have the capability to model ACM and its characteristic feature of fibro-fatty remodelling. The choice of the optimal model has to be based on the experimental project and on the availability of source human samples.

## Methods

### Ethical statement

This study complies with the declaration of Helsinki and was approved by the Centro Cardiologico Monzino Ethics Committee (R1020/19-CCM1072; date of approval: 3/7/2019). Written consent was signed by participating subject. Protocols for stem cell research were approved by the medical ethical committee at Leiden University Medical Center, the Netherlands. The supplementary Tables S1 and S2 summarize the features of Primary and hiPSC-D stromal cell lines, respectively.

### Isolation and culture of Primary cardiac stromal cells

C-MSCs were isolated and cultured as previously reported^6,33^. In brief, ventricular samples were washed with PBS, cut into 2–3 mm pieces, and incubated at 37 °C for 1.5 h under continuous agitation in Iscove’s modified Dulbecco’s media (IMDM; Gibco, Waltham, MA, USA) containing 3 mg/mL collagenase NB4 (Serva, Heidelberg, Germany). The digested solution was then centrifuged at 400 g for 10 min, washed with PBS, and centrifuged again. The obtained pellet was resuspended in growth medium consisting of IMDM supplemented with 20% fetal bovine serum (FBS; Euroclone, Milan, Italy), 10 ng/mL basic fibroblast growth factor (R&D Systems, Minneapolis, Canada), 10, 000 U/mL penicillin (Invitrogen, Carlsbad, CA, USA), 10,000 µg/mL streptomycin (Invitrogen, Carlsbad, CA, USA), and 20 mmol/L L-Glutamine (Sigma-Aldrich, St. Louis, MO, USA). The cells were seeded onto uncoated Petri dishes (Corning, Corning, NY, USA). Non-adherent cells were removed after 24 h.

### Differentiation of hiPSCs into cardiac stromal cells

HiPSCs-D cells were generated from undifferentiated hiPSCs through the induction of cardiac mesoderm followed by an intermediate differentiation into epicardial cells (EPI), as previously described^13^. In brief, 19,800 cells/cm^2^ were seeded on Matrigel at day − 1. On day 0, cardiac mesoderm was induced by BPEL medium (Ng et al., 2008), supplemented with a mixture of cytokines (20 ng/mL BMP4, R&D Systems, Minneapolis, USA; 20 ng/mL ACTIVIN A, (Miltenyi Biotec); 1.5 μM GSK3 inhibitor CHIR99021, Selleckchem). After 3 days, cytokines were removed and the WNT inhibitor XAV939 (5 μM, Tocris, Bristol, UK) was added for 3 days with BMP4 (30 ng/mL) and Retinoic Acid (RA; 1 μM; Sigma Aldrich, St. Louis, MO, USA). On day 6, BPEL medium supplemented with BMP4 (30 ng/mL) and RA (1 μM) was refreshed. On day 9, 14,800 cells/cm^2^ were seeded on plates coated with 5 μg/mL of fibronectin from bovine plasma (fibronectin; Sigma Aldrich, St. Louis, MO, USA) in BPEL medium supplemented with the TGFβ inhibitor SB431542 (10 μM; Tocris, Bristol, UK). By day 12, EPI were confluent and ready to undergo stromal cells differentiation. In brief, 19,800 cells/cm2 EPI were seeded per well on tissue culture plates coated with vitronectin (Gibco) in BPEL medium supplemented with FGF2 (10 ng/ml; R&D Systems, Minneapolis, USA) on day 12. On day 13 and every two days thereafter, medium was refreshed with BPEL supplemented with FGF2 (10 ng/mL). After eight days (on day 21), cells were expanded by switching from BPEL to Fibroblast Growth Medium 3 (FGM3; PromoCell, Heidelberg, Germany). FGM3 was refreshed every 2 days for 8 days in total. After 8 days (on day 29), cells were confluent and ready to be passaged at 1:2 ratio. FGM3 was refreshed the day after passaging and every two days thereafter. Cells were then split at 1:6 ratio approximately twice a week.

### In vitro differentiation of primary and hiPSC-D cardiac stromal cells

To prompt the adipogenic differentiation, hiPSC-D and Primary cells were plated at density of 30,000 cells/cm^2^ and cultured in adipogenic medium, composed of IMDM supplemented with 10% FBS (Euroclone, Milan, Italy), 0.5 mmol/L 3-isobutyl-1-methylxanthine (SigmaAldrich, St. Louis, Missouri, USA), 1 µmol/L hydrocortisone (SigmaAldrich, St. Louis, MO, USA), 0.1 mmol/L indomethacin (Sigma-Aldrich, St. Louis, MO, USA), 10,000 U/mL penicillin (Invitrogen, Carlsbad, CA, USA), 10,000 µg/mL streptomycin (Invitrogen, Carlsbad, CA, USA), and 20 mmol/l L-Glutamine (Sigma-Aldrich, St. Louis, MO, USA) as previously described^33^.

To stimulate pro-fibrotic differentiation, hiPSC-D and Primary cells were plated at density of 30.000 cells/cm^2^ and treated with 5 ng/mL of TGF-β1 (PeproTech, London, UK), after overnight (O/N) growth in low serum GM (2% FBS) as previously described^7^.

To stimulate osteogenic differentiation, hiPSC-D and Primary cells were plated at a density of 10,000 cells/cm^2^ in 6 well-culture plates and cultured in osteogenic medium^34^, composed of DMEM High Glucose (Life Technologies, Paisley, UK) supplemented with 10% FBS (Euroclone, Milan, Italy), 1% penicillin (Invitrogen, Carlsbad, CA, USA), 1% streptomycin (Invitrogen, Carlsbad, CA, USA), 10 mM sodium β-glycerophosphate (Sigma-Aldrich, St. Louis, MO, USA), 0.05 mM ascorbic acid (Sigma-Aldrich, St. Louis, MO, USA). Medium was refreshed three times a week.

For chondrogenic differentiation, hiPSC-D and Primary cells were plated at a density of 10,000 cells/cm^2^ in 6 well-culture plates and cultured in chondrogenic medium, consisting in DMEM High Glucose (Life Technologies, Paisley, UK) supplemented with 1% penicillin (Invitrogen, Carlsbad, CA, USA), 1% streptomycin (Invitrogen, Carlsbad, CA, USA), 100 nM dexamethasone, 10% insulin-transferrin-selenium (ITS-premix) (Life Technologies, Paisley, UK), 1 µg/mL ascorbic acid (Sigma-Aldrich, St. Louis, MO, USA), 1% sodium pyruvate and 10 ng/mL TGF-β1 (PeproTech, London, UK). Medium was refreshed every day.

### Detection of cell differentiation

#### Fibro-adipose differentiation

Cells were fixed using 4% paraformaldehyde (Santa Cruz biotechnology, TX, US) for 10 min. After blocking unspecific binding sites, with PBS supplemented with 5% BSA and 0.1% Triton X-100 (PBS-T/BSA) for 60 min, the slides were incubated with specific primary antibody for Collagen (as reported in Table S4) O/N at 4 °C. Fluorescence-labeled secondary antibodies (Invitrogen, Carlsbad, CA, USA) were added for 1 h at RT. Otherwise, fixed cells were stained using 12.5 ng/mL of Nile Red (Invitrogen, Carlsbad, CA, USA) for 1 h at room temperature (RT). Nuclei were stained with Hoechst 33,342 (Sigma-Aldrich, Saint Louis, MO, USA). Images were acquired with a confocal microscope in Z-stack mode with 40 × oil immersion objective (Zeiss LSM710-ConfoCor3 LSM, Zeiss, Germany) using the software Zen 2008 (Zeiss, Germany). Fluorescence signal quantification was performed using ImageJ software on Z-Stacks images. Single channels from each image were converted into 8-bit grayscale images and thresholded in order to subtract background. The fluorescence value has been normalized to the number of nuclei per field (at least 5 for each experiment). Nuclei counting was performed using the ImageJ tool (National Institutes of Health, Bethesda, MD, USA).

#### Osteo-chondrogenic differentiation

After 10 days, osteogenic or chondrogenic medium was removed and cells were washed three times with PBS (Euroclone, Milan, Italy) and fixed with 4% paraformaldehyde (Santa Cruz biotechnology, TX, USA) for 10 min. Fixed cells were stained using 20 mg/mL of Alizarin Red (Sigma-Aldrich, Saint Louis, MO, USA) or 100 mg/mL of Alcian Blue (Sigma-Aldrich, Saint Louis, MO, USA) for 1 h at RT to assess osteogenic and chondrogenic differentiation, respectively. Images were acquired with inverted tissue culture phase-contrast microscope with 10 × objective (AxioVert 200 M, Zeiss, Germany) using the Axiovision software (Zeiss, Germany).

### Flow cytometry

In order to confirm mesenchymal lineage, cells were incubated with appropriate antibodies (reported in Table S4) and analysed by flow cytometry. Cells were detached with TrypLE Select (Life Technologies, Carlsbad, CA, USA) and incubated with FITC/PE/APC-conjugated antibodies in 100 µL PBS. After washing with PBS to ensure the removal of unbound dye, quantitative results were obtained by evaluating FITC/PE/APC fluorescence with FACS Gallios (Beckman Coulter, Brea, CA, USA). The monoclonal antibodies used in order to confirm mesenchymal lineage and exclude endothelial and hematopoietic origin are the following: CD44, CD90, CD105, CD29, CD14, CD31, CD34, CD45. Immunogenicity of the cells was measured through the marker HLA-DR.

### mRNA extraction and qRT-PCR assay

Cell cultures were lysed in RL lysis buffer (Norgen Biotek corp., Thorold, Canada). RNA was isolated from cells by using a Total RNA Purification kit (Norgen Biotek corp., Thorold, Canada). The quantification of the isolated RNA was determined by NanoDrop spectrophotometer (ND-1000, EuroClone, Milan, Italy). Reverse transcription was conducted with SuperScript III (Invitrogen, Carlsbad, CA, USA) following the manufacturer’s instructions. qRT-PCR was performed with the use of the iQTM SYBR Green Super Mix (Bio-Rad Laboratories, Hercules, CA, USA) and specific primers (reported in Table S5). All reactions were performed in a 96-well format with the 7900HT Fast Real-Time PCR System (Thermo Fisher Scientific, MA, USA). The relative quantities of specific mRNA were obtained with the use of the comparative Ct method and were normalized to the housekeeping gene glyceraldehyde 3-phosphate dehydrogenase (GAPDH).

### Protein extraction and western blot analysis

Cells were lysed in cell lysis buffer (Cell Signaling Technology, Danvers, MA, USA) supplemented with protease and phosphatase inhibitor cocktails (Sigma-Aldrich, Saint Louis, MO, USA). Total protein extracts were subjected to SDS-PAGE and transferred onto a nitrocellulose membrane (Bio-Rad, California, USA). The membranes were opportunely cut for simultaneous blotting with more antibodies based on the protein molecular weight, blocked for 1 h at RT in 5% non-fat dry milk in Wash Buffer (Tris Buffer Sulfate, 0.1% Tween-20) and then incubated O/N at 4 °C with the appropriate primary antibodies (reported in Table S4). The membranes were incubated with peroxidase-conjugated secondary antibodies (GE Healthcare, Chicago, IL, USA) for 1 h. Signals were visualized using the LiteUP Western Blot Chemiluminescent Substrate (EuroClone, Milan, Italy). Images were acquired with the ChemiDoc™ MP Imaging System (Bio-Rad, California, USA), and densitometric analysis of membranes was performed using the ImageJ software (National Institutes of Health, Bethesda, MD, USA). Cell proteins were normalized according to GAPDH or TUBULIN, according to the gel density. The four uncropped original blots are shown in Figs. S2–S5.

### RNA-Seq data pre-processing

Whole-genome transcriptome data were generated using the Illumina Hiseq4000 (100 bp reads). Sequential aligning of raw reads was performed against the GRCh38 (hg38) Human Genome. In particular, reads were aligned to the reference genome using STAR^35^ and Bowtie 2^36^ aligners. Gene annotation and quantification were computed using FeatureCounts software^37^ to obtain a gene expression matrix of raw reads. Raw counts data were then filtered, retaining genes with a minimum of 10 counts in at least 40% of the samples and normalized with the variance stabilizing normalization by the ‘DaMiRSeq’ R package^38^. Person’s correlation analysis and the associated scatterplot have been performed by the ‘Hmisc’ and the ‘ggplot2’ R packages, respectively.

### Pathway analysis

Pathway analysis was performed using the Cytoscape^39^ (v. 3.9.1) plug-in ClueGO (v. 2.5.8)^39^, which estimates the pathways enrichment score on pre-selected set of genes, exploiting a two-sided hypergeometric test. The Gene Ontology^40^ Biological Processes (GO-BP) database was selected as reference. Pathways with less than three associated genes from the uploaded gene list were discharged. Functional related GO terms were grouped by setting a similarity threshold of kappa score of 0.4. Pathways with an associated p-value < 0.05 were deemed as significant.

### Statistical analysis

Continuous variables are presented as mean ± standard error (SEM), and categorical data as counts and proportions. Normally distributed continuous variables normally were compared using the Student t-test for independent samples. Comparisons among four groups were performed with two-way ANOVA test, in association with Tukey’s multiple comparison post-tests. The proportion of the categorical variables was compared using a χ2 analysis or Fisher’s exact test, as appropriate. A p-value < 0.05 was considered statistically significant, except where indicated. Statistical analysis and graphics were produced with GraphPad Prism 9 software.

### Supplementary Information

Supplementary Information.

Supplementary Table.

## Data Availability

The data supporting the findings of this study are available within the article and its Supplementary Materials. All other supporting data are available from the corresponding author on reasonable request. The transcriptomes (ID: GSE233780; GSE234504) data are available on the GEO repository.
