# Supplementary material for: Patient-specific primary and pluripotent stem cell-derived stromal cells recapitulate key aspects of arrhythmogenic cardiomyopathy
Source: Sci Rep. 2023 Sep 27;13:16179. doi: 10.1038/s41598-023-43308-2 (PMC10533531; doi:10.1038/s41598-023-43308-2)
Supplement: Supplementary file 1 — Supplementary Information. [file 41598_2023_43308_MOESM1_ESM.docx]

**Patient-specific primary and pluripotent stem cell-derived stromal cells recapitulate key aspects of Arrhythmogenic cardiomyopathy**

**Angela Serena Maione**1, ***, Viviana Meraviglia**2**, Lara Iengo^1^, Martina Rabino^1^, Mattia Chiesa^3,4^, Valentina Catto**4,5**, Claudio Tondo^5,6^, Giulio Pompilio^1,6^, Milena Bellin2,7,8, Elena Sommariva**1

1Centro Cardiologico Monzino IRCCS, Unit of Vascular Biology and Regenerative Medicine, 20138, Milan, Italy.

^2^Leiden University Medical Center, Department of Anatomy and Embryology, 2333 ZC Leiden, The Netherland.

^3^Centro Cardiologico Monzino IRCCS, Bioinformatics and Artificial Intelligence Facility, 20138, Milan, Italy.

^4^Politecnico di Milano, Department of Electronics, Information and Biomedical Engineering, 20133, Milan, Italy.

^5^Centro Cardiologico Monzino IRCCS, Department of Clinical Electrophysiology and Cardiac Pacing, 20138, Milan, Italy.

^6^Università degli Studi di Milano, Department of Biomedical, Surgical and Dental Sciences, 20122, Milan, Italy.

^7^Department of Biology, University of Padua, 35121 Padua, Italy.

^8^Veneto Institute of Molecular Medicine, 35129 Padua, Italy.

*[angela.maione@cardiologicomonzino.it](mailto:angela.maione@cardiologicomonzino.it)

**Supplementary figures**

**
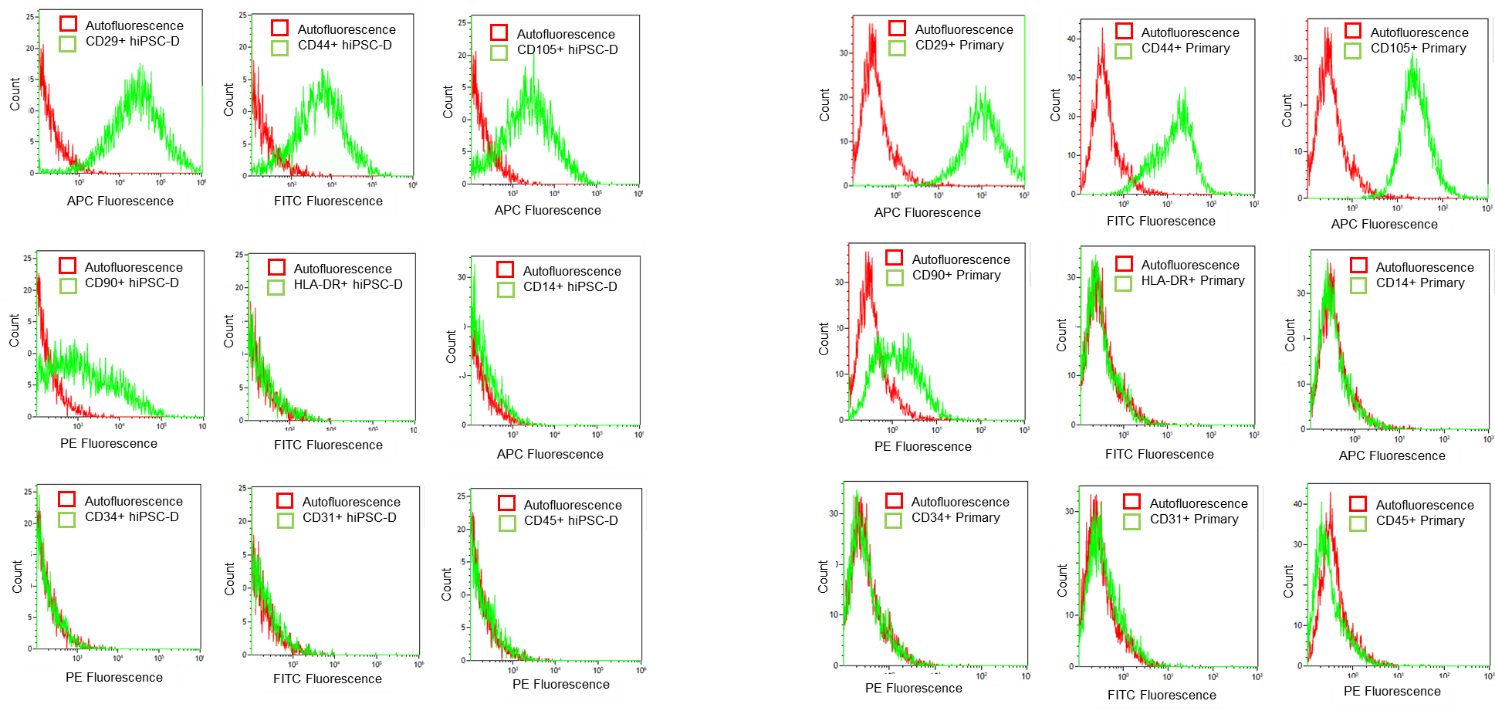
**

**Figure S1.** **FACS plot of mesenchymal markers**

Representative FACS plots of mesenchymal markers analyzed in hiPSC-D (left) and Primary (right) cells.


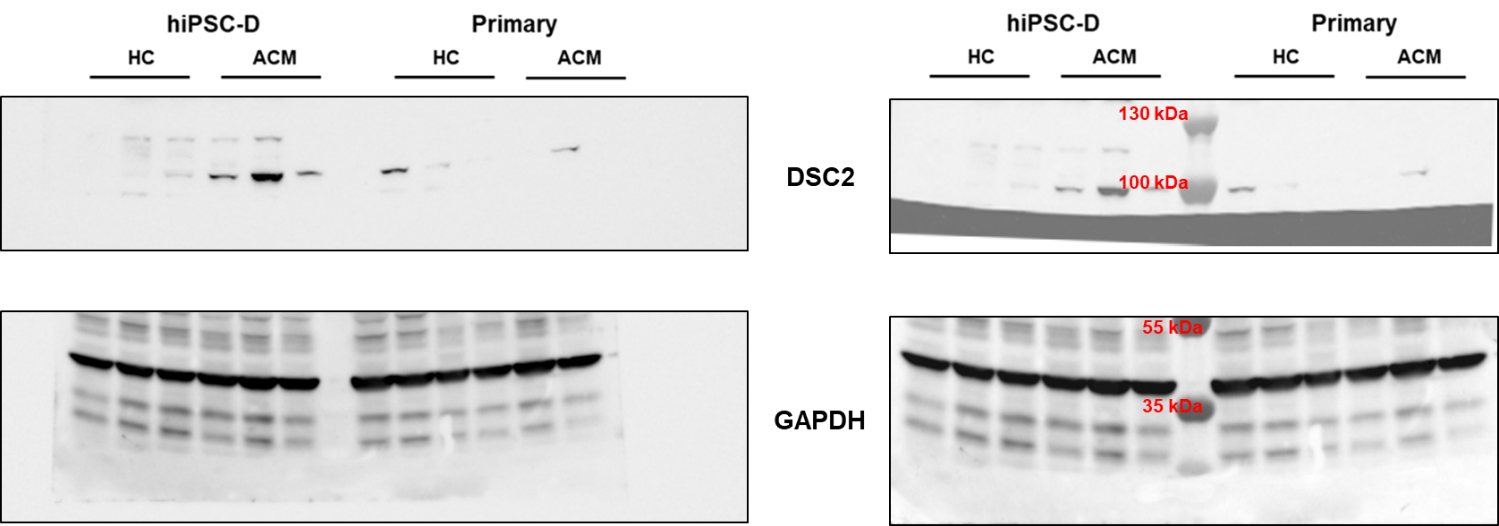


**Figure S2. DSC2 and GAPDH original gels**

Original blots (left) and original blots merged with colorimetric images of the membranes showing the molecular weights (right).

**
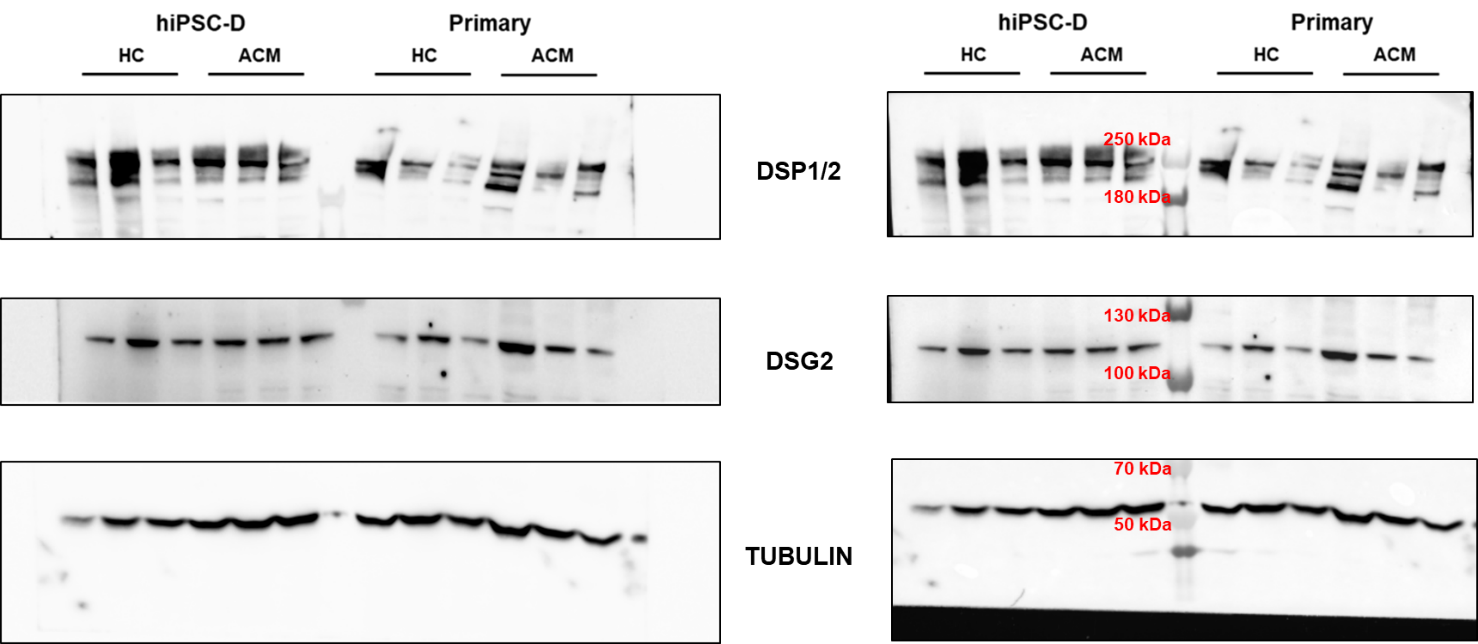
**

**Figure S3. DSP1/2, DSG2 and TUBULIN original gels**

Original blots (left) and original blots merged with colorimetric images of the membranes showing the molecular weights (right).

**
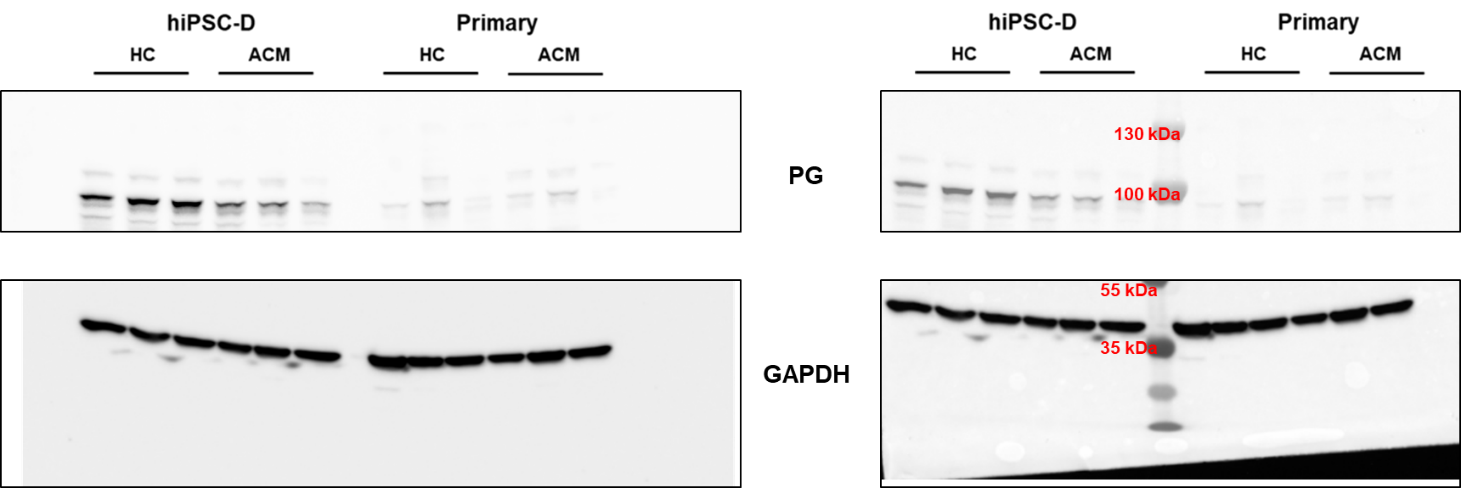
**

**Figure S4. PG and GAPDH original gels**

Original blots (left) and original blots merged with colorimetric images of the membranes showing the corresponding molecular weights (right).


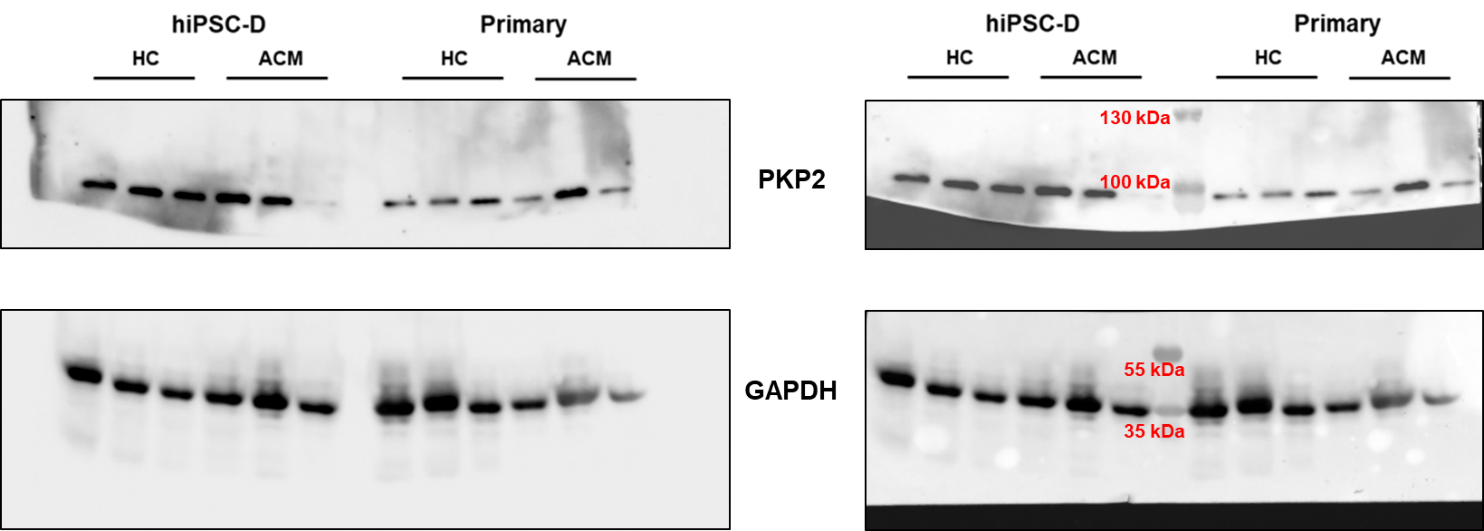


**Figure S5. PKP2 and GAPDH original gels**

Original blots (left) and original blots merged with colorimetric images of the membranes showing the corresponding molecular weights (right).


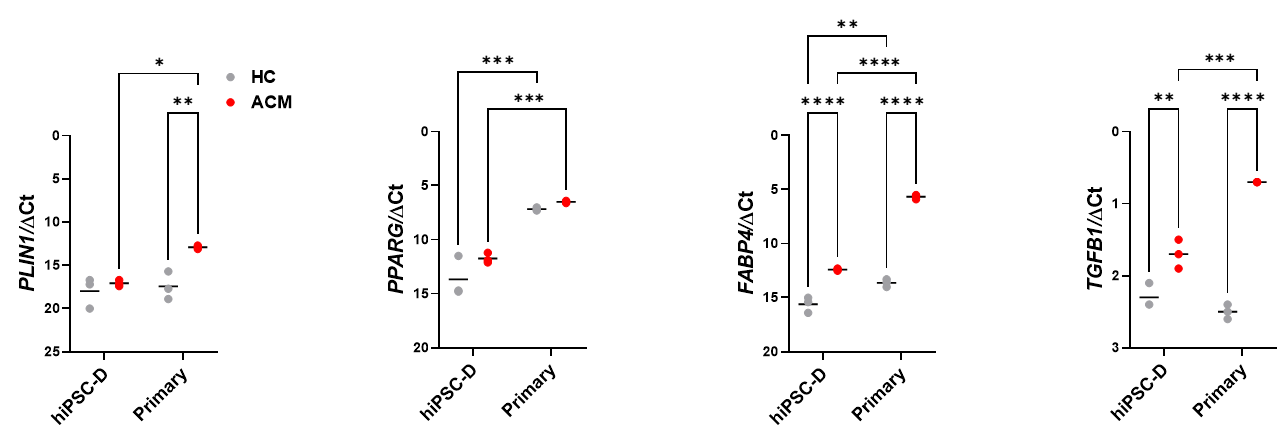


**Figure S6. Analysis of adipogenic and fibrotic markers**

Expression of *PLIN1,* *PPARG,* *FABP4* and *TGFB1* in total RNA extracts of hiPSC-D and Primary cells from HC subjects and ACM patients cultured in adipogenic or pro-fibrotic medium. *GAPDH* was used as house-keeping gene and qRT-PCR data are presented as the genes threshold cycles (Ct) with respect to the housekeeping gene *GAPDH* (ΔCt) (n = 3 replicates; Two-way ANOVA and Tukey’s post-test).


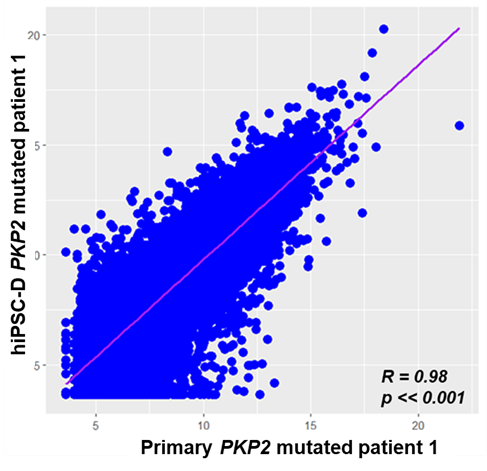


**Figure S7. Transcriptomic analysis of ACM stromal cells.**

Scatter plot showing the relationship between Primary (x-axis) and iPSC-D (y-axis), from ACM patient carrying a PKP2 mutation, gene expression. Blue dots represent a gene, while purple line denotes the correlation trendline.

**Supplementary tables**

| **ACM patients biopsy samples** | | | | | | | | |
| --- | --- | --- | --- | --- | --- | --- | --- | --- |
| **ID** | **Sex/Age**  **(at recruitment)** | **Age/Type of first manifestation** | **Dysfunction/ structural alterations at imaging** | **Tissue characterization** | **Repolarization abnormalities** | **Depolarization conduction**  **abnormalities** | **Arrhythmias** | **Family history/ Genetics** |
| ACM1 | F/ 41 | 27/PVCs | Major | not conclusive | major | negative | minor | major  (*PKP2* c.2013delC p.Lys672ArgfsX12) |
| ACM2 | M/ 42 | 42/VT | Major | not conclusive | major | negative | major | major  (*PKP2* c.1643delG p.Gly548ValfsX15) |
| ACM3 | F/ 52 | 50/PVCs | Major | major | minor | minor | minor | negative |

**Table S1. Summary of bioptic samples collected for Primary stromal cell isolation.**

Clinical data of ACM patients enrolled for biopsy samples. Minor and major scores are given according to the International Task Force Criteria for the diagnosis of ACM (5). VT: ventricular tachycardia; PVCs: premature ventricular contractions. Mutations are reported only when considered pathogenic or likely pathogenic.

| **iPSC line names** | **Gender** | **Age** | **Ethnicity** | **Genotype of locus** | **Disease** |
| --- | --- | --- | --- | --- | --- |
| LUMCi027-A  (<https://hpscreg.eu/cell-line/LUMCi027-A>) | Female | 41 | Caucasian | Heterozygous *PKP2* c.2013delC | Arrhythmogenic Cardiomyopathy |
| LUMC0099iCTRL04 (https://hpscreg.eu/cell-line/LUMCi004-A) | Female | 34 | Caucasian | WT *PKP2* | N/A |

**Table S2. Summary of hiPSC lines.**

**Table S3. Pathways enriched in hiPSC-D or in Primary stromal cells (attached file).**

| **Protein** | **AB** | **Host** | **Company** | **Application/Diluition** |
| --- | --- | --- | --- | --- |
| **PKP2** | polyclonal ab223757 | Rabbit | Abcam | WB; 1:1000 |
| **PG** | (clone 15F11): p8087 | Mouse | Sigma-Aldrich | WB; 1:500 |
| **DSC2** | polyclonal: 610120 | Rabbit | ProgenBiotechnik | WB; 1:500 |
| **DSP1/2** | polyclonal (H-300): sc-33555 | Rabbit | Santa Cruz | WB; 1:500 |
| **DSG2** | monoclonal: 61002 | Mouse | ProgenBiotechnik | WB; 1:50 |
| **GAPDH** | polyclonal (FL-335): sc-25778 | Rabbit | Santa Cruz | WB; 1:1000 |
| **COL1A1** | Monoclonal, #84336 | Rabbit | Cell Signaling | IF; 1:200 |
| **CD29** | Integrin β1-PE (clone MAR4) | Mouse IgG_1_, κ | BD | FACS; 1:100 |
| **CD44** | H-CAM-PE (clone G44-26) | Mouse IgG_2b_, κ | BD | FACS; 1:100 |
| **CD105** | Endoglin-APC (clone 266) | Mouse IgG_1_, κ | BD | FACS; 1:100 |
| **CD90** | THY1-FITC (clone 5E10) | Mouse IgG_1_, κ | BD | FACS; 1:100 |
| **HLA-DR** | HLA-DR-FITC (clone L243) | Mouse IgG_2_, κ | BD | FACS; 1:100 |
| **CD14** | CD14-FITC (MφP9) | Mouse IgG_2b_, κ | BD | FACS; 1:100 |
| **CD34** | Hematopoietic progenitor cell antigen-PE (clone 581) | Mouse IgG_1_, κ | BD | FACS; 1:100 |
| **CD31** | PECAM-1-APC (clone 9G11) | Mouse IgG_1_ | R&D systems | FACS; 1:100 |
| **CD45** | L-CA-PE (clone HI30) | Mouse IgG_1_, κ | BD | FACS; 1:100 |

**Table S4. List of antibodies.**

| **Gene** | **Forward primer** | **Reverse primer** |
| --- | --- | --- |
| ***PKP2*** | TTGGAGGTGGCTGAACTAAATGGG | TTCCACAGCAAACCTGTTATTTGT |
| ***JUP*** | CAGCAATAAGCCTGCCATTGTGGA | CATCCACACTCAGCTGATTCACCA |
| ***DGL2*** | ACAGAGAGGAACACAGCAGCTACA | GCGCGTAACTTCTACGTTGACTTG |
| ***DSP*** | AGGCACCCGGAAGAGAGAATATGA | GCATGGATATCTCCTTGATGGTGG |
| ***DCL2*** | AACCCTCCAATACAGACTGTTCGC | TCATCAATGGTGACCCACCCTGTT |
| ***PLIN1*** | CATTGAGAAGGTGGTGGAGTA | CTTGGCCTTGGGAGACTT |
| ***PPARG*** | ACATAAAGTCCTTCCCGCTGACCA | AAACTGGCAGCCCTGAAAGATGC |
| ***FABP4*** | TTCATACTGGGCCAGGAATTT | TCCATCCCATTTCTGCACAT |
| ***TGFB1*** | AAGTGGACATCAACGGGTTC | GTCCTTGCGGAAGTCAATGT |
| ***GAPDH*** | ATGTTCGTCATGGGTGTGAA | GTCTTCTGGGTGGCAGTGAT |

**Table S5. Primers sequences 5’ - 3’**
